# Healthy Native Youth: Improving Access to Effective, Culturally-Relevant Sexual Health Curricula

**DOI:** 10.3389/fpubh.2018.00225

**Published:** 2018-08-17

**Authors:** Stephanie Craig Rushing, David Stephens, Ross Shegog, Jennifer Torres, Gwenda Gorman, Cornelia Jessen, Amanda Gaston, Jennifer Williamson, Lauren Tingey, Crystal Lee, Andria Apostolou, Carol Kaufman, Christine Margaret Markham

**Affiliations:** ^1^Northwest Portland Area Indian Health Board, Portland, OR, United States; ^2^Center for Health Promotion and Prevention Research, School of Public Health, University of Texas Health Science Center at Houston, Houston, TX, United States; ^3^Inter Tribal Council of Arizona, Inc., Phoenix, AZ, United States; ^4^Division of Community Health Services, Alaska Native Tribal Health Consortium, Anchorage, AK, United States; ^5^Johns Hopkins Center for American Indian Health, Baltimore, MD, United States; ^6^UCLA David Geffen School of Medicine, Semel Institute for Neuroscience and Human Behavior, Global Center for Children and Families, Los Angeles, CA, United States; ^7^Division of Epidemiology and Disease Prevention, Indian Health Service, Rockville, MD, United States; ^8^Colorado School of Public Health, Centers for American Indian and Alaska Native Health, Aurora, CO, United States

**Keywords:** adolescent, sexual health promotion, American Indian and Alaska Native, interventions, dissemination and implementation research, curriculum

## Abstract

Tribal health educators across the United States have found it challenging to locate engaging, culturally-relevant sexual health curricula for American Indian and Alaska Native (AI/AN) youth. Healthy Native Youth is a new online resource that provides a “one-stop-shop” for tribal health advocates to access age-appropriate curricula. The site was designed by a team of advisers representing a diverse group of tribal communities, using a collaborative planning process. The website content and navigation was then refined through usability testing with the target audience. The portal allows users to filter and compare curricula on multiple dimensions, including: age, delivery setting, duration, cost, and evidence of effectiveness, to determine best-fit. It includes all materials needed for implementation free-of-charge, including: facilitator training tools, lesson plans, materials to support participant marketing and recruitment, information about each program's cultural relevance, evaluation methods and findings, and references to publications and reports. The website currently includes mCircle of Life, Native It's Your Game, Native STAND, Native VOICES, and Safe in the Village, among others. Since its launch in August 2016, the site has had over 31,000 page views in all 50 States. The Healthy Native Youth portal provides educators in rural communities a promising new tool to support the dissemination and implementation of evidence-based health curricula in geographically-disbursed AI/AN communities. Lessons learned during the design and dissemination of the Healthy Native Youth website may be of value to other Indigenous populations interested in our approach and our findings.

## Introduction

In the United States (U.S.), the federal government recognizes 573 distinct American Indian and Alaska Native (AI/AN) tribes that represent 2% of population (1). On the whole, the AI/AN population is young, with 30% under 18 years-old; compared to 24% of the total U.S. population (2). As a result, a pressing need exists for health promotion resources in tribal communities that are youth-friendly.

Despite recent declines in teen birth rates in the U.S., racial and ethnic disparities persist (3). AI/AN females 15 to 19 years old have the third highest teen birth rate among all racial/ethnic groups (3) and the highest repeat teen birth rate (4). AI/AN youth are also disproportionately affected by sexually transmitted infections (STIs), including HIV (5, 6).

These health disparities could be ameliorated by implementing culturally-relevant, evidence-based practices (EBPs) (7). To better-align with community assets and reproductive health values, sexual health programs for AI/AN youth should reflect youth's cultural values, learning styles, and traditional coming-of-age teachings (8). To design such programs, teams across the U.S. have been adapting and evaluating sexual health curricula for AI/AN youth across the age spectrum (9–16). This work has largely been done at the local and regional level by Tribal Epidemiology Centers in partnership with universities, with no single national coordinating body.

Consequently, it remains challenging for health educators in disbursed rural AI/AN communities to identify sexual health curricula that have been specifically designed for and rigorously evaluated with Native youth, and many AI/AN communities lack the expertise and resources to adopt, implement, and maintain EBPs (17). To address this need, we designed a website to connect tribal health educators, teachers, and parents to the training and tools needed to select and implement evidence-based curricula. Healthy Native Youth is a “one-stop-shop” for tribal health advocates to access engaging, culturally-relevant, age-appropriate health curricula for AI/AN youth.

The portal allows users to upload and share their own curricula on the website. First Nations communities in Canada and Indigenous Hawaiians have expressed interest in adding their own culturally-tailored resources to the site. Lessons learned during the design and launch of the Healthy Native Youth website may be of interest to other Indigenous communities interested in our approach and our findings.

## The AI/AN adolescent sexual health workgroup

In 2015, a national workgroup was convened by the Northwest Portland Area Indian Health Board, the Alaska Native Tribal Health Consortium, the Inter Tribal Council of Arizona, Inc., and the University of Texas Health Science Center at Houston to support the dissemination of culturally-appropriate sexual health programs to AI/AN youth across the United States. The AI/AN Adolescent Sexual Health Workgroup included tribal health educators and advocates, teachers, school counselors, University partners, and representatives from Tribal Epidemiology Centers, the Indian Health Service, National Indian Health Board, Office of Minority Health, the Bureau of Indian Education, Cardea, UNITY, Big Brothers, Big Sisters, Boys & Girls Club of America Native Services, and other community-based organizations from across the Unites States.

The workgroup met virtually online using AdobeConnect software five times between September 2015 and February 2017 and discussed strategies to engage three priority communication channels: (1) schools, teachers, and educators; (2) parents; and (3) teens and young adults themselves. To reach vastly-disbursed educators and health advocates who work with AI/AN youth, the workgroup determined that offering sexual health curricula and facilitator training tools online could prove useful to support their use of EBPs (17, 18).

The Healthy Native Youth (HNY) website was designed by the workgroup over a 12 month period, guided by collaborative partnerships, usability testing with the target audience, and field testing *in situ*. To inform the content and functionality of the website, a brief online survey was completed by 19 workgroup members and community stakeholders between October 2015 and January 2016. Most respondents indicated that the site should include: age-appropriate sexual health interventions designed for AI/AN youth (*n* = 17), facilitator training videos and guides (*n* = 17), implementation materials (*n* = 16), links to other AI/AN sexual health resources (*n* = 15), sexual health marketing materials (*n* = 11), and a service that connects users to local technical assistance (TA), if needed (*n* = 9). The workgroup then used an iterative mock-up review, discussion, and voting process to select the site's tone and feel, the site's colors and fonts, and the Healthy Native Youth name and logo. Guided by the stakeholder feedback, the workgroup brainstormed, discussed, and refined the site's search and filter criteria, curricula submission and feedback forms, and eligibility criteria for curricula on the site. The Healthy Native Youth website emerged from this year-long collaborative planning process.

## Narrowing digital divide

While a digital divide does affect access to online health resources in some AI/AN communities, those gaps are beginning to narrow (9). “Though infrastructure shortcomings and cost remain concrete barriers—especially with respect to broadband Internet service—AI/ANs have the highest rate of mobile broadband use among minority groups (17).” This is particularly true for young AI/ANs. In 2016, the Northwest Portland Area Indian Health Board surveyed over 675 AI/AN teens and young adults across the U.S. on their media technology use and health information seeking practices and preferences (19). Nearly 78% of youth surveyed had regular access to a smartphone. Over 92% reported accessing the internet from a phone on a daily or weekly basis, and 50% reported going online from a computer as often. Over 62% reported getting health information from the internet on a weekly or monthly basis, and 66% reported getting health information from social networking sites that often. It is based on these trends (and the many advantages of technology-based interventions) that we selected curricula that could be delivered online—like *Multimedia Circle of Life* and *Native It's Your Game*—to adapt and evaluate.

## Website functionality

**Figure 1:**
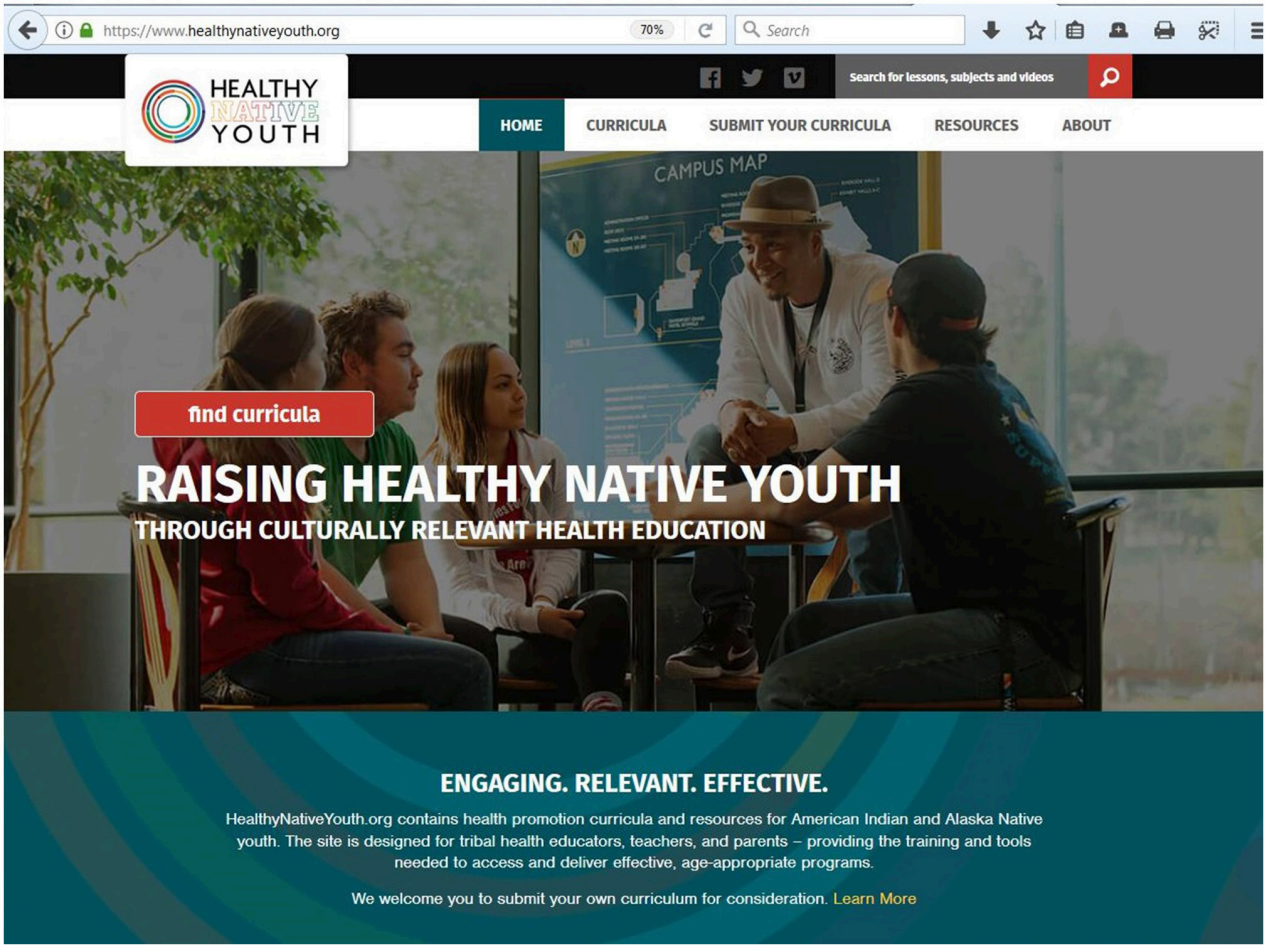
The healthy native youth website.

The www.HealthyNativeYouth.org portal allows users to filter and compare curricula on several dimensions, including by: age, delivery setting, duration, cost, and evidence of effectiveness. For each curriculum included on the site, the website includes all materials needed for implementation, including facilitator training tools, lesson plans, and marketing materials. The website also provides detailed information about: how each program was designed or adapted for cultural relevance; evaluation tools, methods and findings; and references to related publications and reports. To support intertribal sharing, the site also allows users to upload and submit their own health curricula for national distribution (Figure 1).

## Culturally-relevant sexual health curricula

To be included on the website, we sought programs that: (a) promoted positive youth development and healthy decision-making; (b) were purposefully designed or adapted for AI/AN youth or young adults; (c) embraced the cultural teachings and learning styles of AI/AN youth; and (d) have been evaluated with AI/AN youth and demonstrated evidence of effectiveness (ranging from Tribal Best Practices to Emerging Practices). The website currently houses six culturally-relevant sexual health curricula for AI/AN youth:

***Multimedia Circle of Life*** (mCOL) is a sexual risk-reduction program for American Indian youth ages 10–12 years. Seven lessons are delivered online (20 min each), along with 7 group lessons (45 min each), teaching skills related to: goal setting, decision making, and standing up to peer pressure (16). Prevention topics include: how diseases are spread; teen pregnancy; the health effects of HIV/AIDS and sexually transmitted infections; and ways youth can protect themselves.

mCOL was evaluated using a cluster randomized controlled design with 15 Native Boys and Girls Clubs in the Northern Plains (20). At post-intervention and 9-months follow-up, mCOL improved precursors to sexual behavior, including self-efficacy and volition, which may lead to less risky behavior in later years.

***Native It's Your Game*** is a web-based HIV, STI, and pregnancy prevention curriculum for AI/AN youth ages 12–14. Native IYG comprises thirteen 30–50 min interactive lessons, which can be used in the classroom, or as an extracurricular program (8). The program teaches about healthy relationships, life skills, communication, and refusal skills using interactive activities, videos, games, personalized “journaling” activities, tailored feedback, and individually tailored activities. It emphasizes abstinence, but also teaches learners how to protect themselves from pregnancy and sexually transmitted infections using medically accurate information.

Native IYG was adapted and tested as a part of a 4 year randomized control trial funded by the Centers for Disease Control and Prevention (CDC). Twenty five tribal sites agreed to participate in the evaluation, with a total of 523 AI/AN youth aged 12 to 14. Altogether, 402 AI/AN youth completed an online post-intervention survey (76.9% retention) either in the intervention (*n* = 290) or comparison condition (*n* = 112). Five psychosocial variables (of 16 examined) were significantly impacted including increased reasons not to have sex, increased STI and condom knowledge, increased condom availability self-efficacy, and increased condom use self-efficacy (*P* ≤ 0.01) (21).

***Native STAND*** is a comprehensive sexual health curriculum for AI/AN high school students that addresses sexually transmitted infections, HIV/AIDS, and teen pregnancy prevention, while also covering drug and alcohol use, suicide, and dating violence. Twenty-seven sessions support healthy decision-making through interactive discussions and activities that promote diversity, self-esteem, goals and values, team building, negotiation and refusal skills, and effective communication (22). The 1.5 h lessons contain stories from tribal communities that ground learning in cultural teachings.

In 2010, a mixed-methods study was conducted to evaluate Native STAND in four Bureau of Indian Education boarding schools, and from 2010 to 2012, Oregon Health & Science University's Prevention Research Center (OHSU), a Northwest Tribe, and the NPAIHB collaborated to evaluate Native STAND in a tribal Jr High/High School (22, 23). In both trials, teens demonstrated significant improvements in knowledge of STI/HIV prevention, reproductive health, and healthy relationships. Building on that success, OHSU and the NPAIHB recruited 50 tribes and AI/AN organizations to participate in a 5-year *Dissemination, Implementation, and Evaluation* project. The goal of the project is to better understand how tribal communities implement Native STAND and validate program impact.

***Native VOICES*** is a 23-min video, designed to encourage condom use and HIV/STI testing among heterosexual and LGBTQ (Lesbian, Gay, Bisexual, Trans and Queer) American Indian teens and young adults 15–24 years old. The video demonstrates how to negotiate condom use with a partner, and stresses the importance of talking with partners about sexually transmitted infections (24).

In 2014, the NPAIHB partnered with nine tribes across the U.S. to evaluate the effectiveness of the Native VOICES intervention. Together, the sites recruited and consented nearly 800 AI/AN youth 15–24 years old. Changes in participant knowledge, attitude, intention, and behavior were evaluated using pre-, post-, and 6 month follow-up surveys. Youth who watched the video (*n* = 443 respondents) expressed high levels of satisfaction with the Native VOICES intervention. Over 90% felt the video was culturally appropriate for AI/AN people. Over 75% found it to be entertaining or highly entertaining. And 86% felt the characters, scenes, and situations in the video were realistic. The intervention also produced statistically significant improvements in STD knowledge, attitudes toward condoms and dental dams, and self-efficacy toward condoms and dental dams, which were retained at the 6-month follow-up. Native VOICES is the first evidence-based intervention recognized by the CDC for preventing HIV and other STIs among AI/AN youth (25).

***Safe in the Village*** is a video-based intervention designed to start conversations about healthy relationships and safe behaviors with Alaska Native youth. Included is a short movie, actor interviews and a facilitation guide. The movie is a story about Matt, Sarah, and Ben, three friends in rural Alaska navigating life and dealing with peer pressure around relationships, sex, friendships and alcohol. It demonstrates how decisions affect one's future and the importance of having trusted adults and life goals. Safe in the Village was evaluated with 105 youth ages 15–19 years old, who completed pre and post surveys.

***We R Native Teacher's Guide*** is designed to get middle and high school students actively involved in their own health and wellbeing. The guide includes ten 50-min lessons that align to common core standards, utilizing We R Native's multimedia health resources (www.weRnative.org) as a catalyst for group discussion, small group projects, and reflection activities. After participating in the lessons, students will be able to evaluate and support claims while analyzing an online health resource, and will demonstrate their understanding of health topics by designing a community service project.

The curriculum has been reviewed for medical accuracy as part of the Tribal PREP Program within the U.S. Department of Health and Human Services' Family and Youth Services Bureau, and will be evaluated by the Inter-Tribal Council of Michigan, as a Tribal PREP grantee, from 2018 to 2020.

## Website usability test

Once the portal was complete, we conducted a website usability test that took place between September 2016 to February 2017, to refine the site's content and navigation. The team recruited 11 educators who work with AI/AN from across the U.S. to review and provide feedback on their experience selecting and implementing a curriculum on the site.

Usability test participants reported that the website was easy to navigate and included everything they needed to implement the program they selected. They mentioned that the curricula were well received by both Native and non-Native students, and elicited constructive, open discussions. Reviewers also offered ideas to market the site to educators in their communities, and expressed interest in stand-alone lesson plans, in addition to the comprehensive curricula available on the portal. They also discussed curricula that could be added to the site to address other important adolescent health needs, including suicide and coping skills; drug and alcohol use prevention; and trauma informed care.

Many of the suggestions from workgroup members and usability test stakeholders were woven into the final design of the website (i.e., age-appropriate curricula, facilitator training videos and guides, implementation tools, and links to adolescent health resources), working within the available budget and timeline. Other features and functionalities (like interactive maps showing curricula endorsements, and a mechanism to request and receive local technical assistance) will be included in future updates to the site, if funding permits.

## Website analytics and reach

**Figure 2:**
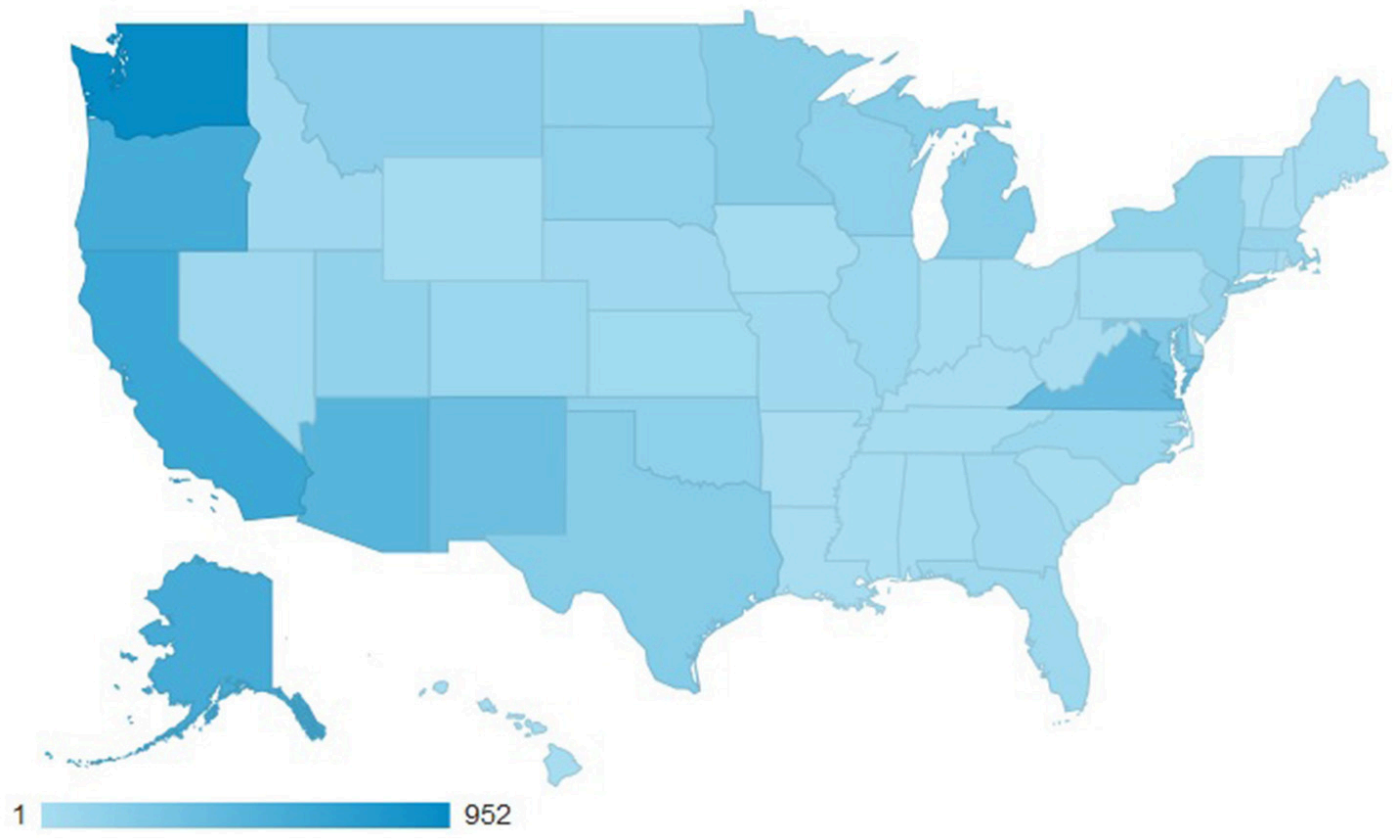
Number of website hits per state, August 2016–April 2018.

Between August 2016 and April 2018, www.HealthyNativeYouth.org had over 31,454 page views and was accessed in all 50 states and 73 countries (Figure 2). Altogether, curricula on the site had over 19,717 page views.

**Figure 3:**
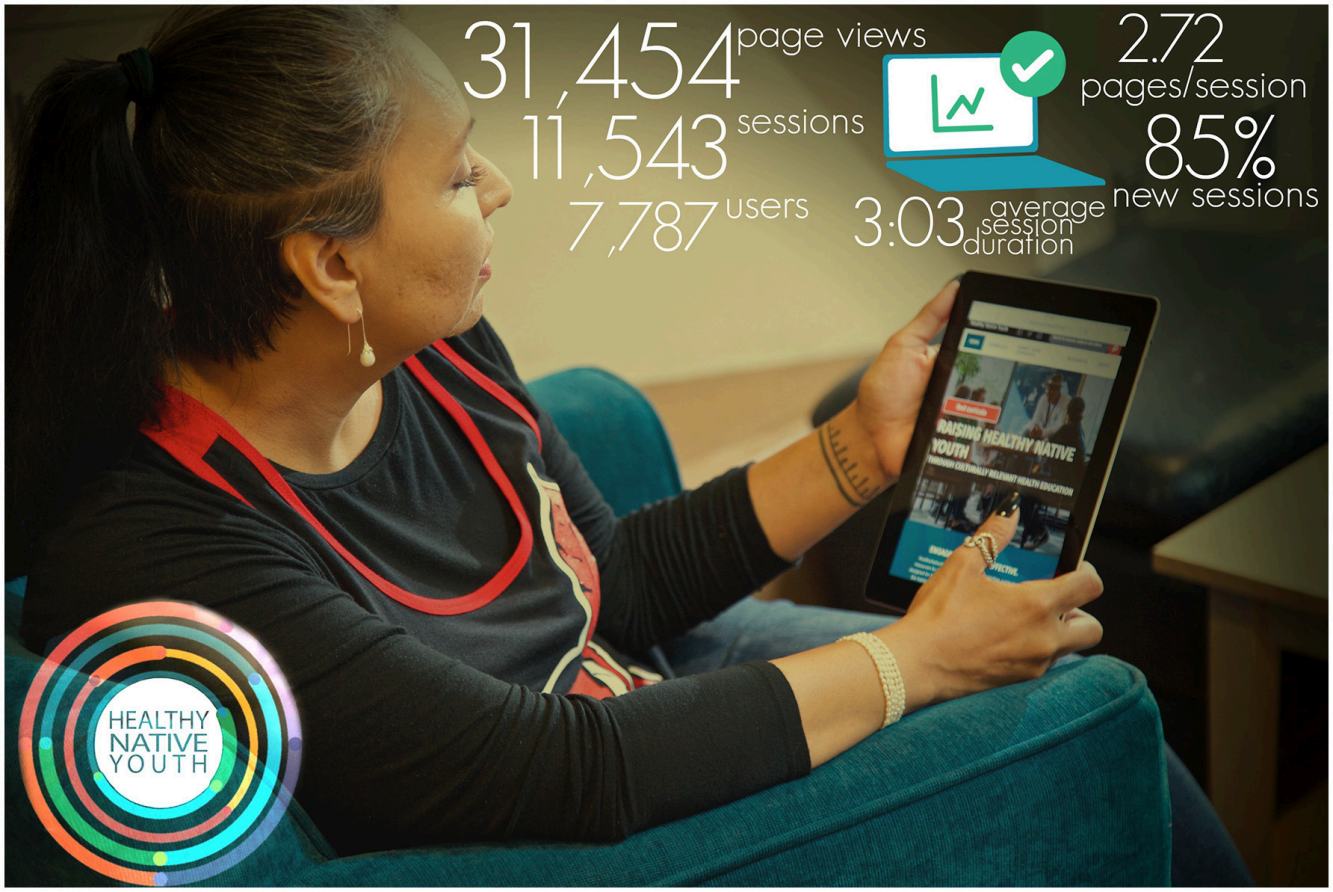
Healthy native youth website analytics, August 2016–April 2018.

People spent an average of 3 min and 3 s on the site, compared to the industry average of 1:22 (Google Analytics: Industry vertical—people & society) (Figure 3). When people visit the site, they typically stay and explore. The site's average bounce rate (percentage of single page visits) is 46.6% vs. the industry average of 62.3%, and the average number of pages visited per session on healthynativeyouth.org is almost 30% greater than the industry average (2.72 vs. 2.14 pages).

There is room for improvement, however. The Healthy Native Youth site performs below the industry average on the total number of sessions (12% lower than the industry average), referral traffic (e.g., visits to www.healthynativeyouth.org from links that appear on a different websites; 41% lower), and social traffic (traffic coming from social networks and social media platforms; 78% lower than the industry average).

## User feedback

To track which communities are using curricula on the website, monitor how many youth are being reached by each program, and collect feedback from educators who have implemented curricula, we designed and included an optional 10-min “Feedback Form” on the site. Those who subscribe to our listerv are prompted twice a year to return to the form to share their feedback.

To date, the feedback form has been submitted by 10 users. Respondents reported implementing the curricula in a variety of settings, including: during school (*n* = 2), after school (*n* = 2), during community events (*n* = 2), at an evening culture class (*n* = 1), at a conference (*n* = 1), and at a Tribal LGBT (Lesbian, Gay, Bisexual, Transgender) youth gathering (*n* = 1).

All of the respondents reported the programs were *somewhat* or *very* engaging for their students. Other comments included:

“*You have done a wonderful job putting this curriculum together. The videos are very helpful.”*

“…I hope our clinic is able to adopt more of this information to share with community members.”

“*I really believe that the modules in the curriculum are great in how they flow in the timeline of adolescent development. I also find that this curriculum is adaptable to the small rural towns that border and neighbor tribal communities; as the cultures for both have strikingly similar barriers to education based on socio-economic status.”*

## Discussion

The Healthy Native Youth website is an exciting new tool to support the dissemination of evidence-based, culturally-relevant health curricula for youth in AI/AN communities.

We recognize that substantial challenges remain, however, that limit curriculum uptake and use in AI/AN communities, and that a website alone is not sufficient to truly maximize program replication and sustainability. Local, regional, and national training and personalized technical assistance will be critical to ensure the successful and repeated use of evidence-based practices accessed through the Healthy Native Youth portal. This network of technical assistance providers exists, but remains un-funded. Tribes and local educators also need seed funding to host kick-off events to build community awareness and buy-in for program implementation and to secure the necessary approvals from local Tribal governing bodies.

The AI/AN Adolescent Sexual Health Workgroup continues to convene quarterly to monitor website usage and reach. Despite lower referral and social traffic compared to industry benchmarks, the number of direct new user sessions is 32% higher than the industry standard, likely due in part to our active print and word-of-mouth marketing. The workgroup also brainstorms strategies to increase the number of EBPs available on the site, and plans—in the next year—to create resources for parents to support parent-child communication about sexual health and market HNY's sexual health resources directly to AI/AN youth.

## Limitations and strengths

Our formative survey, usability test, and curricula feedback forms involved small sample sizes that may not be representative of all AI/AN educators in the U.S. who have used curricula available on the site. The design and functionality of the website was strengthened however, by involving diverse stakeholders in its design, including tribal representatives, adolescent health educators, researchers, and federal agencies.

## Conclusion

Given existing disparities in sexual and reproductive health experienced by many Indigenous youth, the Healthy Native Youth website holds great promise as a means to empower educators with the resources and tools needed to promote healthy decision-making. Lessons learned during the design and dissemination of the Healthy Native Youth website may be of value to other Indigenous populations interested in our approach and our findings.

## Ethics statement

This study was carried out in accordance with the recommendations of the Institutional Review Board at the University of Texas Health Science Center at Houston, Committee for the Protection of Human Subjects (CPHS). The protocol was approved by the CPHS. The CPHS determined that this project was not human subjects research, but a Quality Improvement project, therefore no informed consent was required.

## Author's note

Those interested can sign up to receive curricula updates in the red footer at the bottom of the www.HealthyNativeYouth.org homepage. You can also follow us on Facebook to receive news and resources supporting AI/AN adolescent health: www.facebook.com/HealthyNativeYouth.

## Author contributions

As members of the AI/AN Adolescent Sexual Health Workgroup, all authors contributed to the design of the Healthy Native Youth website and critically reviewed and approved this manuscript.

### Conflict of interest statement

The authors declare that the research was conducted in the absence of any commercial or financial relationships that could be construed as a potential conflict of interest.
